# Alterations of Fat and Ketone Body Metabolism in ALS and SMA—A Prospective Observational Study

**DOI:** 10.1111/ene.70132

**Published:** 2025-04-09

**Authors:** C. Herrmann, Z. Uzelac, S. Michels, A. Weber, L. Richter, Z. Elmas, L. Jagodzinski, C. Wurster, J. Schuster, J. Dreyhaupt, J. Dorst

**Affiliations:** ^1^ Department of Neurology University of Ulm Ulm Germany; ^2^ Institute for Epidemiology and Medical Biometry Ulm Germany; ^3^ German Center for Neurodegenerative Diseases (DZNE) Ulm Germany

**Keywords:** amyotrophic lateral sclerosis, fatty acids, ketone bodies, metabolic alterations, spinal muscular atrophy

## Abstract

**Background:**

Amyotrophic lateral sclerdosis (ALS) and spinal muscular atrophy (SMA) are motor neuron diseases associated with distinct metabolic alterations. ALS patients feature an increased resting energy expenditure (REE) causing weight loss and cachexia. In SMA, a disturbed utilization of free fatty acids has been described. These metabolic alterations negatively affect prognosis in both diseases. The objective of this study was to further characterize these changes to identify potential therapeutic targets.

**Methods:**

Between 11/2020 and 08/2022, 112 ALS patients, 77 SMA patients, and 50 controls were recruited in the Department of Neurology of Ulm University. Standardized blood and urinary samples were collected to analyze fat and ketone metabolism.

**Results:**

Ketone body levels were higher in ALS and SMA compared to controls. In both diseases, patients with higher BMI featured higher ketone bodies and free fatty acids compared to those with lower BMI, while in controls we found the opposite phenomenon. In SMA, more severe disease types were associated with higher ketone body levels. Compared to ALS, SMA patients featured higher ketone body and free fatty acid levels.

**Conclusions:**

Our data suggest that already during early disease stages, ALS patients produce ketone bodies to compensate for the energy deficit. In SMA, on the other hand, the persistence of ketogenesis may indicate an upregulation of all available metabolic pathways for energy production due to the disturbance of fatty acid utilization. Therefore, the application of additional sources of energy, such as ketone bodies, might constitute a promising therapeutic option in both diseases.

## 1 Introduction

Amyotrophic lateral sclerosis (ALS) and spinal muscular atrophy (SMA) are both classified as motor neuron diseases. However, metabolic changes also seem to play an essential role in pathogenesis. While ALS is a fast‐progressing, mainly sporadic neurodegenerative disease including degeneration of upper and lower motor neurons, SMA is a hereditary neuromuscular disorder characterized by degeneration of alpha motor neurons in the spinal cord, leading to progressive muscle wasting and paresis.

In ALS, a significant energy deficit causes weight loss in the majority of patients and is known as an independent negative prognostic marker [1, 2]. Studies using indirect calorimetry have shown that resting energy expenditure (REE) was approximately 14% higher compared to healthy subjects in about 62% of the study population, depending on age, sex, and type of onset [3]. Mitochondrial alterations, specifically an impaired function of complex I in the respiratory chain, lead to insufficient adenosine triphosphate (ATP) production and contribute to the energy deficit [4, 5]. Interestingly, indirect calorimetry in healthy presymptomatic gene carriers unexpectedly showed a lower REE compared to healthy controls [6], possibly indicating a compensatory saving of energy in presymptomatic stages, while neurofilament light chain (NfL) serum levels were still normal and no motor symptoms were present. Thus, metabolic changes occur early, highlighting their pathophysiological importance as well as their significance as a promising biomarker and therapeutic target.

In ALS, high‐caloric interventions aiming at compensating for the energy deficit have shown beneficial effects [7, 8, 9]: In a recent placebo‐controlled, multicenter, randomized controlled trial (LIPCAL‐ALS), a high‐caloric, high‐fat dietary supplement (+405 kcal/day, 100% fat in addition to normal food intake) in 201 patients induced a significant survival benefit for fast‐progressing patients [7]. In the same subgroup, loss of body weight was less extensive in the intervention group compared to the placebo group [10]. These positive clinical findings were accompanied by a significant decline of NfL serum levels [8].

In SMA, multiple studies have demonstrated impairments in fatty acid metabolism: In both the patients and murine models, non‐alcoholic fatty liver disease, dyslipidemia, and defects of several enzymes involved in beta‐oxidation, the primary pathway for lipid metabolism, have been observed [11, 12, 13]. Impairment of mitochondrial beta‐oxidation results in a shifting of fatty acids to omega‐oxidation as a “recue pathway”, which is located in the endoplasmic reticulum and cytosol. Finally, dicarboxylic acids are produced, which can undergo further metabolism or be excreted in the urine. In patients with SMA, analysis of urine samples revealed the presence of dicarboxylic acids, indicating a defect in fatty acid metabolism, especially beta‐oxidation [13, 14]. However, contrary to ALS, these findings have not yet prompted subsequent nutritional studies.

In this study, we aimed to further investigate the metabolic changes in ALS and SMA to identify possible biomarkers as well as therapeutic nutritional targets.

## 2 Methods

### 2.1 Study Population

Between 11/2020 and 08/2022, 112 ALS patients, 77 SMA patients, and 50 controls were recruited in the Department of Neurology of Ulm University, Germany. Included ALS patients were 18 years or older with possible, probable, or definite ALS according to the revised El Escorial criteria [15]. SMA patients had a genetically confirmed SMA. Since the study was conducted in the field of adult neurology, the majority of patients were diagnosed with SMA type 2 or 3. Control patients had other neurological diseases, such as cerebral ischemia or multiple sclerosis, excluding neurodegenerative diseases and other diseases that are known to be accompanied by metabolic alterations. Also, patients with known metabolic disorders, for example, diabetes mellitus, were excluded. The study was approved by the local ethics committee of Ulm University, application numbers 397/20 and 511/20, and was conducted in accordance with the Declaration of Helsinki. All study participants gave written informed consent.

### 2.2 Measures

Blood and urinary samples were collected between 10 and 12 a.m. The following parameters were analyzed: ketone bodies (beta‐hydroxybutyrate and acetoacetate in serum, acetone in urine), free fatty acids, carnitine (only in ALS and controls), total cholesterol, high‐density lipoprotein (HDL), low‐density lipoprotein (LDL), and triglycerides.

Serum for NfL analysis was obtained from peripheral blood by centrifugation (800 g, 5 min) and stored within 2 h at −80°C. Blood NfL concentrations were measured with the commercially available kits for the ELLA microfluidic system (BioTechne, Minneapolis, USA). The interassay coefficients of variation (CV) were < 15%.

The ALS functional rating scale revised (ALSFRS‐R) slope pre‐baseline was calculated according to the formula: (48‐ score at screening visit)/(months between onset and screening visit).

### 2.3 Statistics

For descriptive analysis, median and interquartile range are given. For all group comparisons, the Mann–Whitney *U* test was used. To estimate the effects of BMI and NfL on metabolic parameters, we performed a median split and patients were classified as high/low BMI and high/low NfL accordingly. To analyze the effect of age and sex, multiple linear regression models were applied.

All statistical tests were performed at a two‐sided significance level of alpha ≤ 0.05. As this was an exploratory study, no adjustments for multiple testing were made, and accordingly, all results were interpreted as hypothesis‐generating. Statistical analyses were done with GraphPad Prism and SAS.

## 3 Results

### 3.1 Baseline Characteristics

Baseline characteristics are shown in Table 1. Although age was similar in ALS (median: 65 years) and the control group (median: 66 years), SMA patients were younger (median: 30 years). BMI was higher in the control group (median: 26.0 kg/m^2^) compared to ALS and SMA patients (ALS: 22.9 kg/m^2^, SMA: 22.5 kg/m^2^). Sex distribution was similar in all three groups.

**TABLE 1:** Baseline characteristics.

	ALS	SMA	Control
Number of subjects	112	77	50
Age (median (IQR))	65 years (25–70)	30 years (21–49)	66 years (57–73)
Sex			
Male	53 (47%)	43 (56%)	29 (58%)
Female	59 (53%)	34 (44%)	21 (42%)
BMI (median (IQR))	22.9 kg/m^2^ (19.7–26.5)	22.5 kg/m^2^ (17.0–27.6)	26.0 kg/m^2^ (24.2–28.5)
ALS onset			
Bulbar	29 (26%)		
Spinal	83 (74%)		
ALSFRS‐R slope pre‐baseline (median (IQR))	0.84 points/month (0.52–1.33)		
NfL serum levels	129.0 pg/mL (76.3–205.3)		
SMA type			
1		8 (10%)	
2		32 (42%)	
3		34 (44%)	
4		3 (4%)	
Treatment			
Nusinersen		52 (77%)	
Risdiplam		15 (23%)	
Diagnosis			
Cerebral ischemia			19
MS			7
CIDP			9
Other autoimmune diseases			7
Other			8

Abbreviations: ALS, amyotrophic lateral sclerosis; BMI, body mass index; CIDP, chronic inflammatory demyelinating polyneuropathy; MS, multiple sclerosis; SMA, spinal muscular atrophy.

### 3.2 Ketone Bodies, Fatty Acids, and Carnitine

**FIGURE 1:**
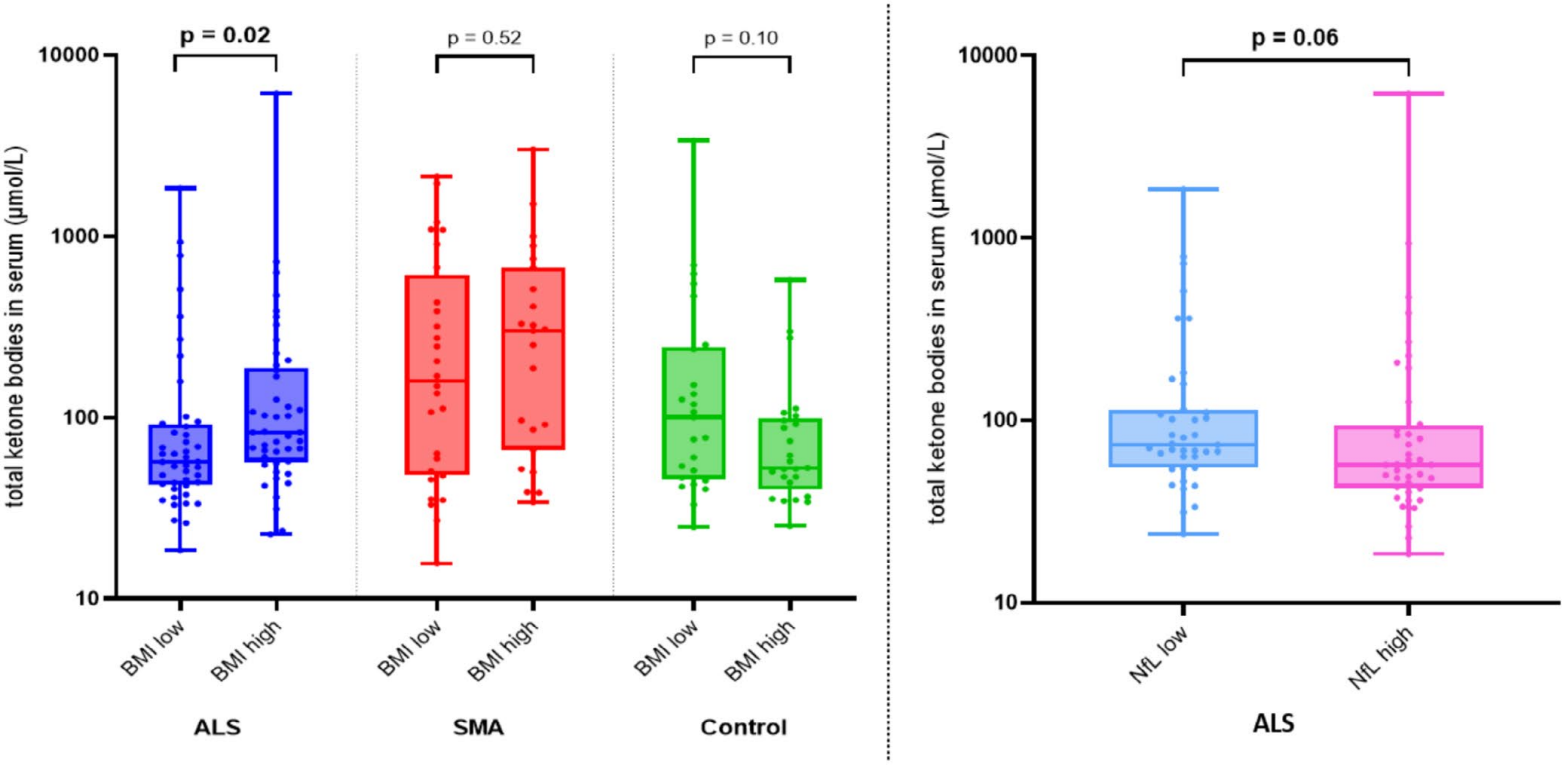
Total ketone body levels in relation to BMI and NfL in ALS, SMA, and controls. Left: In ALS and SMA, ketone bodies were higher in patients with higher BMI, while subjects in the control group showed the opposite trend. Right: In ALS, a trend towards higher ketone bodies was found in patients with lower NfL serum levels. ALS, amyotrophic lateral sclerosis; BMI, body mass index; NfL, neurofilament light chain; SMA, spinal muscular atrophy.

In ALS, higher BMI was associated with higher ketone body levels (*p* = 0.02; Figure 1). While the median of ketone body levels in the lower BMI group was 56.95 μmol/L (IQR 42.50–92.03), patients with a higher BMI had a median of 83.00 μmol/L (IQR 56.70–188.15). Levels of individual ketone bodies, beta‐hydroxybutyrate and acetoacetate, were also higher in the group with higher BMI (both: *p* = 0.04). Moreover, patients with lower NfL levels, which are associated with slower disease progression, showed higher ketone body levels (73.40 μmol/L (IQR 54.90–114.60) vs. 56.95 μmol/L (IQR 42.50–93.68)), although this result was not significant (*p* = 0.06), and ketone body levels showed a significant overlap in both groups. Thus, higher ketone body levels were more prevalent in patients with higher energy stores (higher BMI) and less aggressive courses of disease (lower NfL; Figure 1).

Interestingly, the control group showed the opposite phenomenon regarding total ketone body levels in relation to BMI: Subjects with higher BMI showed a trend towards lower ketone body levels (52.80 μmol/L (IQR 40.25–99.60)) compared to those with lower BMI (100.40 μmol (IQR 45.70–245.55); *p* = 0.10). Beta‐hydroxybutyrate and acetoacetate showed the same trend as total ketone body levels.

**FIGURE 2:**
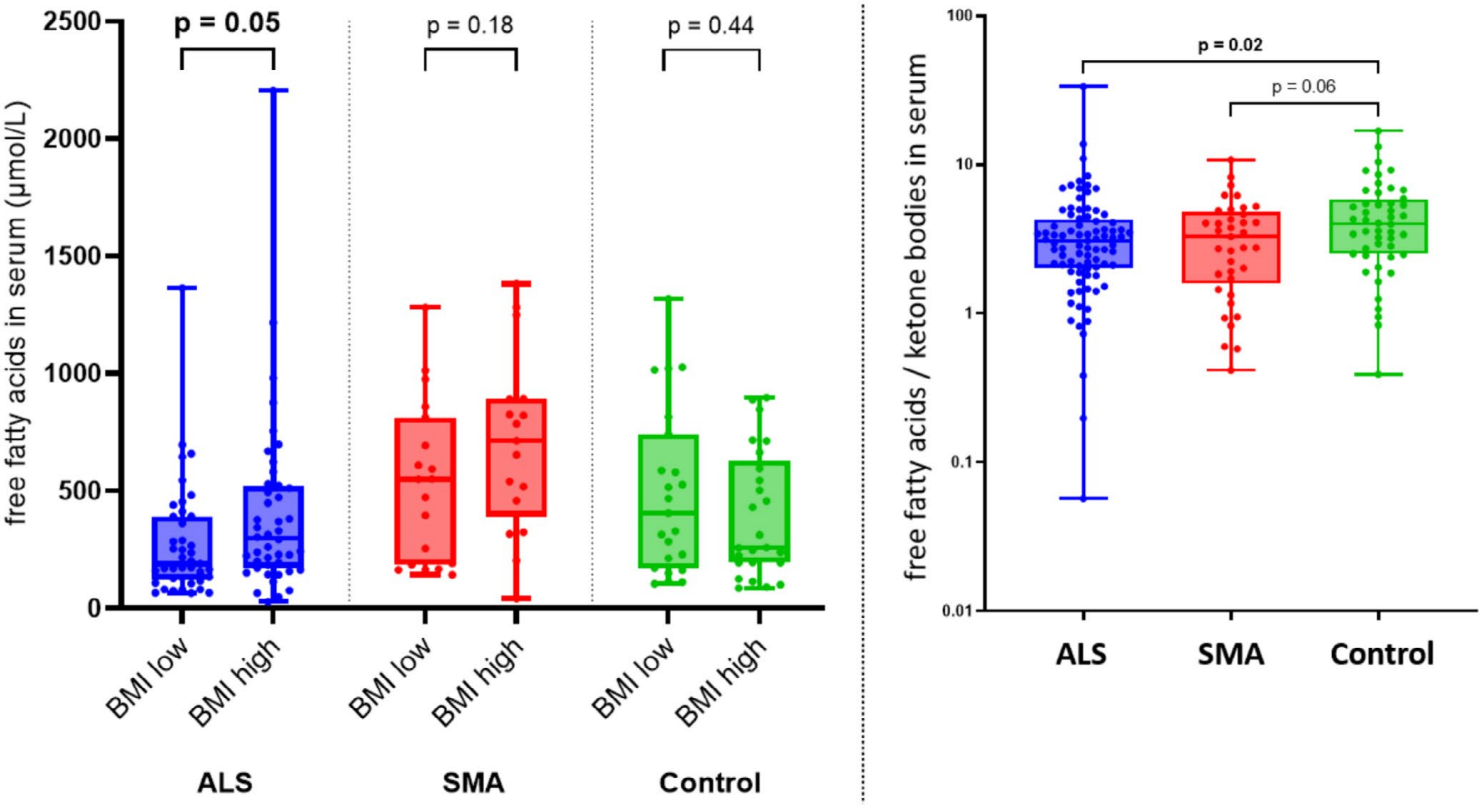
Free fatty acids in relation to BMI and ketone bodies in ALS, SMA, and controls. Left: In ALS and SMA, free fatty acids were higher in patients with higher BMI. Controls showed a contrary pattern with higher free fatty acids in patients with lower BMI. The results for SMA and controls were not statistically significant. Right: The ratio of free fatty acids to ketone bodies was lowest in ALS, followed by SMA, while controls showed the highest ratio. ALS, amyotrophic lateral sclerosis; BMI, body mass index; SMA, spinal muscular atrophy.

As expected, higher levels of free fatty acids were found in patients with higher BMI in the ALS cohort (297.95 μmol/L (IQR 174.03–519.80) vs. 189.90 μmol/L (IQR 122.20–390.95), *p* = 0.05) (Figure 2). Carnitine, which is required for the transport of fatty acids into the mitochondria, was also higher in ALS patients with higher BMI (*p* = 0.02). In the control group, there was no similar correlation between BMI, fatty acids, and carnitine levels. Of note, ALS patients showed lower free fatty acids compared to the control group (238.4 μmol/L (IQR 153.4–451.4) vs. 321.5 μmol/L (IQR 194.5–646.6), *p* = 0.04). This finding was accompanied by a trend towards lower levels of carnitine (7.2 μmol/L (IQR 6.1–8.6) vs. 8.3 μmol/L (IQR 6.5–9.6), *p* = 0.08).

We also analyzed the relationship between free fatty acid and ketone body levels to determine the proportion of fatty acids converted to ketone bodies. The ratio of free fatty acids/ketone bodies was lower in ALS patients than in the control group (3.08 (IQR 2.02–4.26) vs. 4.01 (IQR 2.50–5.82); *p* = 0.02), indicating that ALS patients produce more ketone bodies from fatty acids compared to controls.

The SMA cohort showed some similarities to ALS: A higher BMI was also associated with higher total ketone body levels, although not statistically significant. However, total ketone body levels were higher in SMA (196.3 μmol/L (57.6–551.8) compared to ALS (68.10 μmol/L (IQR 47.15–120.4), *p* < 0.001) and controls (68.1 μmol/L (IQR 44.6–128.3), *p* = 0.002), although this effect was no longer significant in multivariate analysis (*p* = 0.53) including age and sex as additional influencing variables. These findings were accompanied with higher levels of free fatty acids in SMA (592.6 μmol/L (IQR 286.2–842.4)) compared to ALS (238.4 μmol/L, IQR 153.4–451.4), *p* < 0.001) and controls (321.5 (IQR 194.5–646.5), *p* = 0.03), which was confirmed by multivariate analysis (*p* = 0.02) (Figure 2).

**FIGURE 3:**
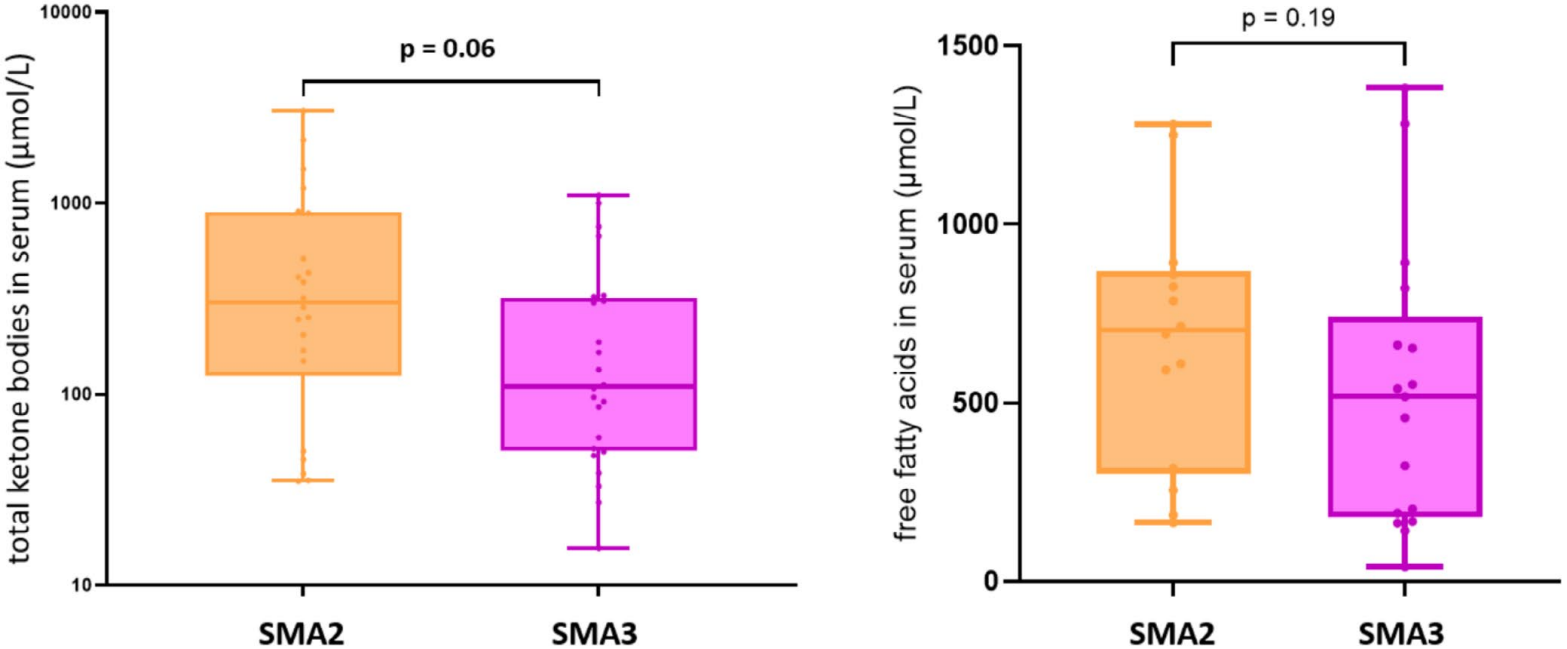
Total ketone bodies and free fatty acids in SMA2 and SMA3. SMA, spinal muscular atrophy.

Compared to SMA3, patients with SMA2 showed a tendency toward higher serum levels of ketone bodies and free fatty acids, indicating a correlation between disease severity and a more pronounced defect in fatty acid metabolism (Figure 3).

### 3.3 Triglycerides

**FIGURE 4:**
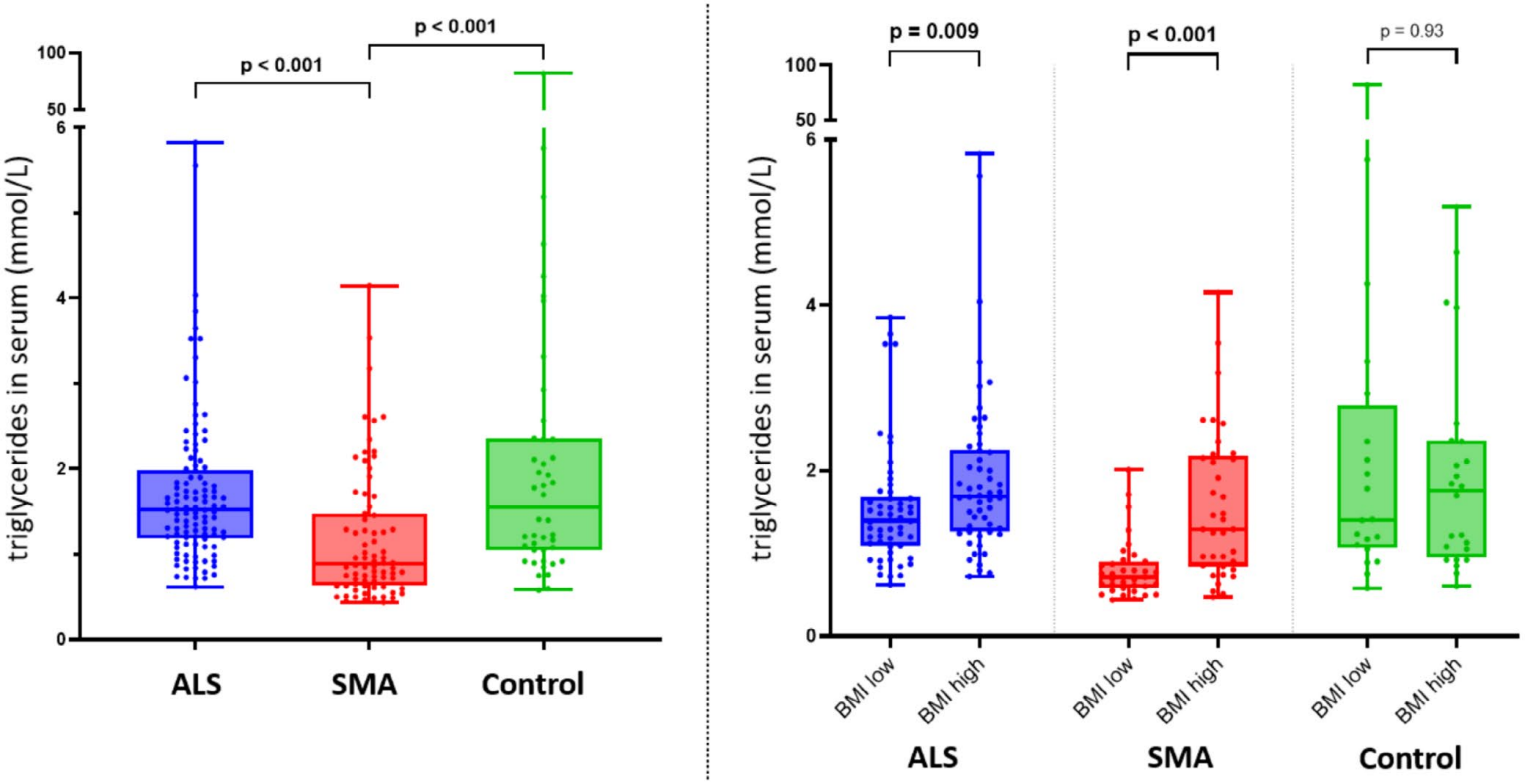
Triglyceride levels in ALS, SMA and controls. Left: In SMA, triglycerides were lower than in ALS and controls. Right: In patients with higher BMI, triglycerides were higher compared to patients with lower BMI. ALS, amyotrophic lateral sclerosis; BMI, body mass index; SMA: spinal muscular atrophy.

Triglycerides are known as a positive prognostic marker in ALS. Consistently, in the ALS cohort, patients with lower NfL levels (indicating slower disease progression) had higher triglyceride levels. However, overall, no significant difference in triglyceride levels was observed between ALS and controls (*p* = 0.77). Patients with SMA exhibited lower triglyceride levels (0.9 mmol/L (IQR 0.6–1.5)) than controls (1.6 mmol/L (IQR 1.1–2.4), *p* < 0.001) and ALS patients (1.5 mmol/L (IQR 1.2–2.0), *p* < 0.001) (Figure 4), which might be partially explained by the younger age of SMA patients as revealed by multivariate analysis, in which this result was no longer significant (*p* = 0.13). In both ALS (*p* < 0.001) and SMA (*p* < 0.001), but not in the control group, a higher BMI was associated with higher triglyceride levels (Figure 4).

### 3.4 Cholesterol

**FIGURE 5:**
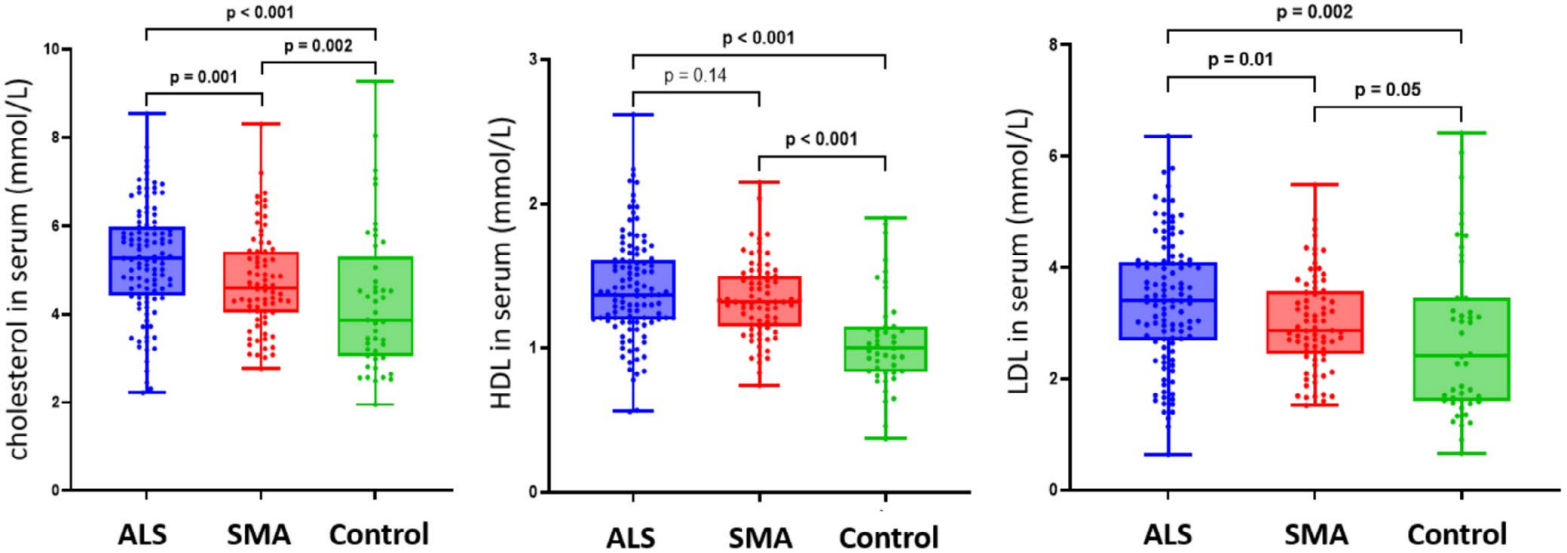
Cholesterol, HDL, and LDL levels in ALS, SMA, and controls. Cholesterol, HDL, and LDL levels were the highest in ALS, followed by SMA and controls. ALS, amyotrophic lateral sclerosis; SMA, spinal muscular atrophy.

Total cholesterol, HDL, and LDL also showed the highest levels in ALS, which was confirmed by multivariate analysis (*p* < 0.001 for all variables) (Figure 5).

## 4 Discussion

Our data revealed significant disparities in lipid and ketone metabolism between individuals with motor neuron diseases and controls. In our study, levels of ketone bodies, free fatty acids, and carnitine were higher in ALS patients with higher BMI compared to those with lower BMI. Notably, in healthy controls, the opposite phenomenon was observed. In SMA, more severe SMA types were associated with higher levels of ketone bodies and free fatty acids.

Ketone bodies are primarily produced in the mitochondria of liver cells, but are also synthesized by astrocytes to a lesser extent [16]. Physiologically, the most potent stimuli for ketogenesis include glucose restriction, fasting, and ketogenic diet, which refers to a high‐fat, low‐carbohydrate nutrition. In healthy individuals, ketone bodies are physiologically generated during sleep or extensive exercising [17].

Based on previous studies, we anticipated higher levels of ketone bodies in patients with lower BMI compared to those with higher BMI [18]. In the control group, this hypothesis was confirmed, as ketone body levels were higher in leaner patients. Surprisingly, however, ketone body levels in ALS showed opposite tendencies, as ketone bodies were higher in patients with higher BMI. What may initially seem implausible can be explained by the presence of hypermetabolism in a large proportion of patients with ALS. Recent studies have shown that metabolic changes occur before the onset of motor symptoms [6], suggesting the presence of hypermetabolism at a very early stage. Consequently, the organism initiates the mobilization of all potential compensatory substrates aiming at generating supplementary energy. Studies in an isolated working rat heart have shown that the generation of ATP from ketone bodies is more efficient compared to glucose and fatty acids [19]. The ratio of free fatty acids/ketone bodies was lower in ALS than in controls, suggesting that free fatty acids are metabolized to more energetic ketone bodies rather than being directly utilized in beta‐oxidation. As the disease progresses, triglyceride stores may be depleted gradually, resulting in a reduced ability to produce ketone bodies with decreasing BMI. Eventually, energy stores become exhausted at a certain point during the course of the disease.

When comparing free fatty acids and carnitine levels in ALS to controls, we found that both substrates were lower in ALS. Similar results were observed in a previous study with 30 ALS patients [20]. Lower levels of free fatty acids and carnitine may be associated with decreasing BMI and loss of muscle mass, as carnitine is mainly stored in muscle tissue. In the control group, no differences in free fatty acids and carnitine were found between subjects with higher and lower BMI, indicating that these substrates are relatively unaffected by BMI. In contrast, ALS patients with higher BMI had higher free fatty acid and carnitine levels compared to patients with lower BMI. Assuming a compensatory use of free fatty acids and carnitine for energy production in ALS, lower levels of these substrates in patients with lower BMI may indicate an increasing depletion of energy stores with decreasing BMI.

Additionally, within the ALS cohort, we observed higher ketone body levels in patients with a slower disease course, as indicated by lower NfL levels. NfL, an essential component for the structural integrity of myelinated axons, is highly elevated in ALS due to neuronal and axonal degeneration. Intriguingly, preclinical trials have demonstrated that ketone bodies protect myelin‐forming oligodendrocytes, thereby reducing axonal damage [21]. This possibly suggests that patients with higher ketone bodies may experience less axonal damage as reflected by lower NfL levels. However, this result was not statistically significant (*p* = 0.06), and there was a significant overlap of ketone body levels in both groups. With these limitations in mind, these findings may point to the potential of a ketogenic diet as a molecular strategy for ALS.

In SMA, our data demonstrated increased ketone body and free fatty acid levels and (like in ALS) a lower ratio of free fatty acids to ketone bodies compared to controls. Several studies have identified defects in fatty acid metabolism in SMA resulting in an increased excretion of organic acids and acyl‐carnitines, but intact ketogenesis [11, 13, 14, 22, 23, 24, 25]. Similarly, our data showed increased serum ketone bodies, indicating a pronounced ketone metabolism. Physiologically, fatty acids undergo beta‐oxidation. When beta‐oxidation is impaired, fatty acids increasingly enter omega‐oxidation, resulting in the production of dicarboxylic acids, which are excreted in the urine. Acetyl‐CoA, the product of beta‐oxidation, is used in the citrate cycle, which produces energy substrates (e.g., NADH^+^H^+^) to generate ATP in the respiratory chain. Alternatively, acetyl‐CoA may be metabolized to ketone bodies. The underlying mechanism of persistent ketogenesis in the context of disrupted fatty acid metabolism in SMA is still unclear. We assume that an accumulation of fatty acids and acetyl‐CoA may exceed the capacity for further metabolization. Since potential energy should not be wasted, all available metabolic pathways may be upregulated, resulting in additional ketogenesis. Another explanation could be the lower REE [26] due to the loss of metabolically active cells, which might cause a lower consumption of ketone bodies and therefore increased serum levels.

Moreover, ketone body levels appear to be related to the severity of SMA, as type 2 showed higher ketone body levels than type 3. These findings are consistent with those of previous studies in children with SMA, in which abnormalities in fatty acid metabolism were more pronounced in more severe forms of SMA [13, 23].

Triglycerides are a compound of fatty acids and glycerol that serve as the primary energy storage. In the event of energy restriction, for example, due to fasting, triglycerides are released from adipose tissue, subsequently entering beta‐oxidation and finally producing ATP. Our data are in line with recent studies showing increased triglycerides in ALS [27, 28]. Elevated serum triglyceride levels may indicate increased energy requirements, which may result in the release of triglycerides from adipose tissue. Higher triglyceride levels were associated with lower NfL levels, confirming recent studies that attributed a protective effect to higher triglyceride levels [27]. In SMA, triglyceride levels were lowest, indicating that they are stored in adipose tissue rather than circulating in the blood.

Cholesterol is primarily utilized for forming lipid membranes in body cells, but it is also involved in the production of steroid hormones and bile acids. Highest levels were observed in individuals with ALS, followed by SMA and controls. Recent publications have highlighted an association between increased cholesterol levels and both the risk of ALS as well as faster disease progression [29, 30], which seems to apply to total cholesterol, HDL, and LDL. Increased degradation of motor neurons, muscle tissue, and other body cells in both ALS and SMA may result in the release of cholesterol from neuronal lipid membranes into the bloodstream. Since ALS features a faster disease progression than SMA, elevated cholesterol levels may be attributed to the increased rate of cellular turnover. In ALS, intracellular aggregates of TDP‐43, which are involved in the regulation of cholesterol biosynthesis [31], might constitute another explanation.

In conclusion, metabolic processes observed in the early stages of ALS seem to mirror the organism's efforts to mobilize all available energy sources, including ketone bodies, fatty acids, and triglycerides, to compensate for the energy deficit. In later stages of the disease, these energy reserves are depleted, resulting in a reduction of fatty acids and ketone bodies. This hypothesis is in line with the known protective effect of higher BMI, as patients with higher fat reserves can support these compensatory mechanisms for a longer time. It is important to note that both different phenotypes of ALS as well as the presence of non‐invasive ventilation have been shown to influence REE [3]. For example, in thoracic onset ALS, increased respiratory workload has been shown to cause increased REE and, consequently, severe weight loss [32]. However, due to the limited number of subjects in this study, we were not able to conduct any meaningful subgroup analyses for distinct phenotypes. Therefore, further research is needed in this regard. In SMA, our findings corroborate the hypothesis of disrupted lipid metabolism, which appears to be more pronounced in severe disease patterns. These findings have significant clinical implications, as nutritional interventions may provide alternative or additional energy substrates.

In ALS, a high‐caloric, high‐fat dietary supplement (+405 kcal/d) has already been shown to provide beneficial effects regarding survival, BMI, and NfL serum levels in fast progressing patients (LIPCAL‐ALS‐study) [7, 8]. A placebo‐controlled randomized study with the 1.5‐fold dosage of the same intervention (+630 kcal/d) is currently ongoing (LIPCAL‐ALS II‐study, NCT06280079). As an alternative therapeutic approach, additional oral ketone bodies as a tool to provide high‐energy substrates are being administered in another placebo‐controlled randomized study (KETO‐ALS‐study, NCT04820478).

In SMA, nutritional interventional studies are still scarce. Based on our results, the administration of a carbohydrate‐rich diet might constitute a promising approach to bypass the defective fatty acid metabolism. However, randomized controlled clinical studies are needed to prove this hypothesis.

## Author Contributions

**C. Herrmann:** conceptualization, methodology, data curation, validation, investigation, formal analysis, visualization, writing – original draft. **J. Dreyhaupt:** writing – review and editing, methodology, formal analysis, data curation. **J. Schuster:** writing – review and editing. **C. Wurster:** writing – review and editing. **J. Dorst:** conceptualization, methodology, data curation, formal analysis, validation, supervision, visualization, writing – original draft. **A. Weber:** writing – review and editing, investigation. **S. Michels:** writing – review and editing. **Z. Uzelac:** writing – review and editing. **L. Jagodzinski:** writing – review and editing, investigation. **Z. Elmas:** writing – review and editing. **L. Richter:** writing – review and editing.

## Conflicts of Interest

The authors declare no conflicts of interest.

## Data Availability

Individual participant data that underlie the results reported in this article, after de‐identification (text, tables, and figures) will be available. Data will be available beginning 3 months and ending 5 years following article publication. Data will be shared with researchers who provide a methodologically sound proposal. Data will be shared for analyses to achieve the aims in the approved proposal. Proposals should be directed to christine.herrmann@uni-ulm.de; to gain access, data requestors will need to sign a data access agreement.
